# QTN mapping, gene prediction, and simulation breeding of four-seed pod numbers in soybean

**DOI:** 10.3389/fpls.2025.1614971

**Published:** 2025-07-25

**Authors:** Ming Yuan, Xu Sun, Zhiyuan Yu, Haoyue Sun, Sheng Dong, Jie Zhang, Bo Hu, Wen-Xia Li, Hailong Ning, Wencheng Lu

**Affiliations:** ^1^ Heilongjiang Academy of Agricultural Science, Qiqihar, China; ^2^ Key Laboratory of Soybean Biology, Ministry of Education, Key Laboratory of Soybean Biology and Breeding/Genetics, Ministry of Agriculture, Northeast Agricultural University, Harbin, Heilongjiang, China; ^3^ Heilongjiang Academy of Agricultural Sciences, Heihe, China

**Keywords:** soybean, number of four-seed pods, quantitative trait nucleotide, candidate genes, breeding schemes

## Abstract

The number of four-seed pods (NFSP) in soybean is an important yield trait and a quantitative trait regulated by multiple genes. Mapping quantitative trait nucleotide (QTN), mining major genes, and screening excellent breeding schemes for NFSP are of great significance for breeding high-yielding soybean varieties. In this study, a germplasm population (GP) containing 455 soybean varieties was planted in five environments to investigate NFSP. Single-nucleotide polymorphism (SNP) genotype data were obtained based on the Axiom_SoyaSNP 180K chip, and a genome-wide association study (GWAS) was used to locate QTNs, which were used to predict candidate genes and simulate breeding. The results showed that there was genetic variation in NFSP and different gene expression in various environments. The broad-sense heritability of NFSP over multiple environments was 72.7%. A total of 89 QTNs correlated with the number of four pods were identified on 20 chromosomes, including 34 stable QTNs repeatedly detected by multiple methods or in multiple environments and four QTNs with an additive by environment interaction effect. In the decay regions of 34 stable QTNs, three genes related to NFSP in soybean were screened and verified by haplotype analysis, namely, Glyma.13G105400, Glyma.04G063700, and Glyma.04G063100. Multiple regression analysis of 89 QTNs on NFSP was used to establish molecular-assisted selection models for five environments, which could explain 44.82%–55.06% of the phenotypic variation in NFSP. Based on this model, 153 breeding schemes were selected for five environments, which could achieve the breeding goals of NFSP over 43, 43, 25, 31, and 40 under 21AC, 21XY, 23WD, 23QQHE, and 23AC environments, respectively. These results laid the foundation for understanding the genetic mechanism of pod formation in soybean and molecular breeding of high-yielding varieties.

## 1 Introduction

Soybean [*Glycine max* (L.) Merr.] is rich in protein and oil and is widely used in food, feed, environmental protection, medicine, and other fields. The breeding of high-seed-yield varieties is always an important goal of soybean breeding. Pod number is one of the important yield components, and it also has different degrees of promoting or inhibiting effects on other traits. Among various pod traits, the number of four-seed pods (NFSP) is a potential factor affecting yield and one of the key targets of soybean germplasm selection for high yield (Ning et al., 2018; Liu et al., 2019).

To improve the breeding efficiency of pod number, linkage analysis and genome-wide association study (GWAS) were used to map the quantitative trait locus (QTL) and quantitative trait nucleotide (QTN) associated with NFSP. Currently, 117 QTLs and 154 QTNs for pod and seed number-related traits have been listed in the SoyBase website (www.soybase.org). In addition, Vieira et al. (2006) detected two QTLs for pod number per plant using a recombinant inbred line (RIL) population. Liu et al. (2019) used a population of four-way RIL population to map 76 QTLs related to pod number, containing 23 NFSP QTLs. Li et al. (2021) constructed chromosome segment substitution lines (CSSLs) and fine-mapped an NFSP QTL on chromosome 7, which was narrowed to 321 kb. Zheng et al. (2022) used this population to localize seven, two, and six QTLs associated with the ratio of two-, three-, and four-seed pod number, respectively. Cao et al. (2023) used these CSSLs to detect 22 QTLs controlling the ratio of four-seed pod number.

The development of the soybean pod is affected by a variety of regulatory pathways, among which the calcium signaling pathway and the abscisic acid pathway have been found. Studies have shown that abscisic acid secreted by soybean roots can control early pod expansion during the critical period of 3–5 days after flowering (Liu et al., 2004). Abscisic acid in the seed or pod can affect early pistillate abortion by inhibiting cell division (Dembinska et al., 1992; Setter and Flannigan, 2001; Liu et al., 2003; Yang et al., 2020, 2017; Zhang et al., 2021). The first identified gene controlling NFSP is Ln, which affects pod formation by controlling early flowering cell division and expansion (Jeong et al., 2012; Fang et al., 2013; Sayama et al., 2017). Liu et al. (2015) found that the development of four-seed pods is associated with a complex network involving multiple physiological and metabolic pathways. Fang et al. (2021) found that a differentially expressed gene, Glyma.10G089300, encodes a 5-kDa photosystem II protein, which is involved in the energy conversion of photosynthesis. Four differentially expressed genes (DEGs) encoded calproteins and a WRKY transcription factor—Glyma.10G089300, Glyma.10G089300, Glyma.10G002200, Glyma.09G280200, and Glyma.04G136200—and played a significant role in the plant–pathogen interaction pathway, which suggested that the proportion of NFSP in above-ground plants may be affected by the action of underground roots. In addition, they identified two pectin-related DEGs (Glyma.13G060900 and Glyma.13G064700) that overlapped with a major QTL on chromosome 13 controlling four fruit size traits, which may be important candidate genes affecting the proportion of NFSP.

Traditional breeding is conducted according to the determination of phenotypes in field experiments. However, the limitations of traditional breeding are a long breeding cycle and environmental influences, which lead to poor accuracy and low efficiency. Simulation breeding based on molecular breeding knowledge can avoid the blindness of traditional breeding, improve breeding efficiency, and reduce cost by predicting the breeding value of target traits under different parental cross combinations and breeding methods in a specific environment. At present, there are relatively few studies on crop simulation breeding because there are few simulation breeding platform software available for systematic analysis. Blib is a multi-module simulation platform that can handle more complex genetic effects and models (Zhang et al., 2022). One of the main and unified application modules is *in silico* breeding (ISB), which can be used to study simulated breeding programs for plant clones, pure lines, and hybrids (Li et al., 2025).

Summarizing the above studies, it can be seen that there are problems in the related studies on pod number traits. First, the breeding traits are mostly focused on the total pod number per plant, and there are few in-depth studies on two-pod number, three-pod number, and NFSP. Second, most of the molecular marker techniques are based on the simple sequence repetition (SSR) framework maps to map QTLs. The low marker density of genetic maps is not conducive to subsequent gene mining and molecular marker-assisted selection studies (Tischner et al., 2003; Sun et al., 2006; Vieira et al., 2006; Chen et al., 2007; Palomeque et al., 2009, 2010; Li et al., 2008; Zhang et al., 2010; Jeong et al., 2011; Liu et al., 2011; Ragin et al., 2012; Kuroda et al., 2013; Shim et al., 2017; Liu et al., 2019). Compared with linkage analysis, genome-wide single-nucleotide polymorphism (SNP)-based association analysis is easier to comprehensively and deeply understand the genetic basis of breeding traits (Fang et al., 2017; Hao et al., 2012; Hu et al., 2014; Qi et al., 2014; Zhang et al., 2015).

In this study, the multi-locus random-SNP effect mixed linear model (mrMLM) and 3VmrMLM methods were first applied to analyze the GWAS of NFSP in a germplasm population in five environments. Based on the detected QTNs, candidate genes were predicted, and the optimal breeding schemes were selected. This study provided a theoretical basis and technical support for understanding the genetic basis of soybean pod formation and the genetic improvement of high-yielding soybean cultivars.

## 2 Materials and methods

### 2.1 Genetic material

The germplasm population (GP) containing 455 soybean varieties previously constructed by our research group, including four landraces, 387 domestic varieties, and 44 foreign varieties (Li et al., 2020), was used as the genetic material for QTN localization, candidate gene prediction, and simulated breeding studies (**Supplementary Table S1**).

### 2.2 Field experiment design and trait investigation

GPs were planted in five environments, namely, in Acheng (E126.95°, N45.52°) in 2021 (21AC), Xiangyang (E126.94°, N45.74°) in 2021 (21XY), Acheng (E126.95°, N45.74°) in 2023 (23AC), Qiqihar (E123.92°, N47.35°) in 2023 (23QQ), and Wudalianchi (E126.21°, N48.52°) in 2023 (23WD). A field experiment was conducted using a completely randomized block design with three replications. The plot contained three rows with a length of 5 m and a row spacing of 0.67. The details of planting density, fertilizer application, and sowing date for each environment are listed in **Supplementary Table S2**. Field management was consistent with local soybean production conditions. At the maturity stage, 10 plants per variety were randomly selected to investigate NFSP, and the average value was used as the phenotypic data for NFSP of each variety.

### 2.3 Phenotypic variation analysis

The mean, standard deviation, minimum, maximum, range, kurtosis, skewness, and coefficient of variation were calculated for the population phenotypic data. The generalized linear model method was used to conduct an analysis of variance with the variation sources of block, environment, genotype, and genotype × environment interaction effect. The mixed linear model method was used to calculate the variance component, and the heritability was estimated based on the variance component. The following formula was used to calculate the multi-environment generalized heritability of the GP.


$$h^{2}=\sigma_{G}^{2}/\left(\sigma_{G}^{2}+\sigma_{GE}^{2}/E+\sigma_{e}^{2}/ER\right)$$

where $h^{2}\;,\sigma_{G}^{2}\;,\;\;\sigma_{GE}^{2},\;\sigma_{e}^{2}\;,\;\;E$ , and R represent broad-sense heritability, genetic variance, genotype × environment interaction variance, error variance, number of environments, and number of blocks, respectively.

The above statistical analysis was performed using SAS 9.2 (SAS Institute, Cary, NC, USA).

### 2.4 Genome-wide association analysis

In previous studies, the Axiom^®^_SoyaSNP 180K chip has been used to identify SNP genotypes in the population, and 109,676 SNP markers have been obtained. By structural analysis, the population was divided into two subgroups, comprising 132 and 323 individuals, respectively. The attenuation distance was determined to be 86K by analysis of linkage disequilibrium (Li et al., 2020).

Seven multi-locus genetic model methods were used to realize the GWAS of soybean NFSP, including the mrMLM (Wang et al., 2016), fast multi-locus random-effect mixed linear model (FASTmrMLM) (Tamba et al., 2017), fast multi-locus random-SNP-effect efficient mixed model association (FASTmrEMMA) (Wen et al., 2018), polygenic-background-control-based least angle regression plus empirical Bayes (pLARmEB) (Zhang et al., 2017), iterative sure independence screening EM-Bayesian LASSO algorithm (ISIS EM-BLASSO) (Tamba et al., 2017), integration of Kruskal–Wallis test with empirical Bayes (pKWmEB) (Ren et al., 2018), and IIIVmrMLM (Li et al., 2022a; Li et al., 2022b).

The above analysis processes were implemented through two R software packages: mrMLM.GUI (https://cran.r-project.org/web/packages/mrMLM.GUI/index.html) and 3VmrMLM (https://github.com/YuanmingZhang65/IIIVmrMLM). The parameters of the R software were set as default values: the critical value of the p-value in the first stage of mrMLM, FASTmrEMMA, pLARmEB, pKWmEB, and ISIS EM-BLASSO was set to 0.01, and the critical value of the p-value of the FASTmrEMMA method was set to 0.005. In the second stage, the critical value of the log of odd (LOD) value of the R software was set to 3, and the critical value of the p-value was set to 0.0002. Likelihood=“REML”, SearchRadius=100, CriLOD=3, SelectVariable=143, and Bootstrap=FALSE. The parameter settings for the IIIVmrMLM method were the same as those for the preceding method.

### 2.5 Prediction of NFSP-related candidate genes

First, all the genes in the Phytozome website (https://phytozome-next.jgi.doe.gov) were downloaded from the linkage disequilibrium region of the mapped stable QTNs (43 kb upstream and downstream of the QTN site). Then, pathway analysis was conducted using the Kyoto Encyclopedia of Genes and Genomes (KEGG) (https://www.kegg.jp), and the candidate genes related to NFSP were preliminarily predicted based on gene function annotation and relevant literature review.

### 2.6 Haplotype analysis

A resequencing analysis was conducted on 202 germplasm resources in the GP group, and the NFSP phenotypic data were measured. The target gene sequences were extracted using the Vcftools software (https://vcftools.github.io/downloads.html), and the site variations were analyzed to classify the allelic variations. Combined with the NFSP phenotypic data of soybeans, the haplotype analysis of the non-synonymous mutation variation sites in the coding region was performed using the Haploview software (https://www.broadinstitute.org/haploview/downloads#JAR).

### 2.7 Construction of molecular marker-assisted selection model and simulation breeding

The two SNP genotypes of each significant QTN were assigned −1 and 1, and then the molecular marker-assisted selection (MAS) models of 89 QTNs for NFSP were established by multiple regression analysis. The superior allele of each QTN was determined based on the effect size of each QTN. When the effect value of QTNs was positive, the genotype encoding 1 was considered the superior allele. Conversely, when the effect value was negative, the genotype coding −1 was the superior allele. For each QTN, the percentage of superior alleles in all individuals was the ratio of the number of varieties containing superior alleles to the total number of varieties. For each variety, the proportion of superior alleles was calculated by dividing the number of superior alleles by the total number of QTNs. The molecular MAS model was constructed using the SAS 9.2 software (SAS Institute, Cary, NC, USA).

**Figure 1:**
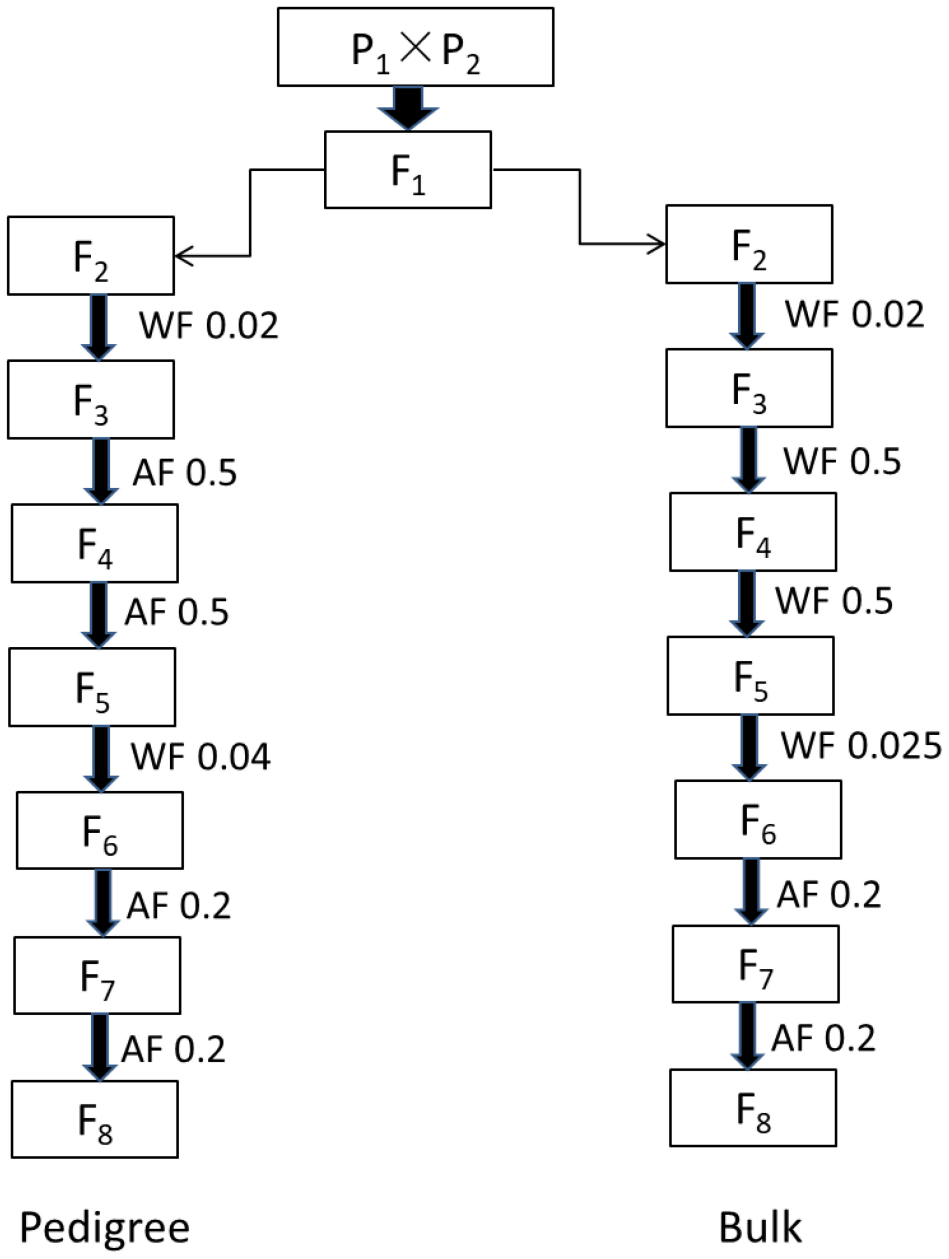
Flowchart of simulating breeding scheme treated by single-cross combination pedigree (top) and bulk (bottom) selection methods. “Pedigree” represents the individual harvest of selected plants within a family, while “bulk” represents the mixed harvest of selected plants within a family. “AF” represents selection among families, and “WF” represents selection within families. The decimal numbers represent the selection ratio, and in each generation, families with the highest phenotypes were selected according to the given ratio. For F1, 10 plants of each hybrid were planted; for F2, 200/500/800 plants of each line were planted; for F3 and F4, 30 plants of each line were planted; and for F5–F7, 50 plants of each line were planted.

To achieve the goal of improving NFSP, hybrid combinations capable of aggregating superior QTN allelic variations were screened. According to the design of a half-diallel cross, 455 parents were paired two by two, resulting in a total of 103,285 hybrid combinations, with single crosses being conducted. After hybridization, the modified pedigree method (Ped) and the bulk selection method (Blk) were applied. The F2 generation was set with population sizes of 200, 500, and 800 individuals. Therefore, each hybrid combination had six breeding schemes, namely, Ped200, Ped500, Ped800, Blk200, Blk500, and Blk800. The population sizes for F1, F3, F4, F5, F6, and F7 were 10, 30, 30, 50, 50, and 50 plants, respectively. Inter-generation selection was adopted in the F2, F4, F6, and F7 generations, while intra-generation selection was used in the F5 generation. The process of each breeding scheme followed the procedure shown in **Figure 1**.

## 3 Results and analysis

### 3.1 Phenotypic variation in population NFSP

**Figure 2:**
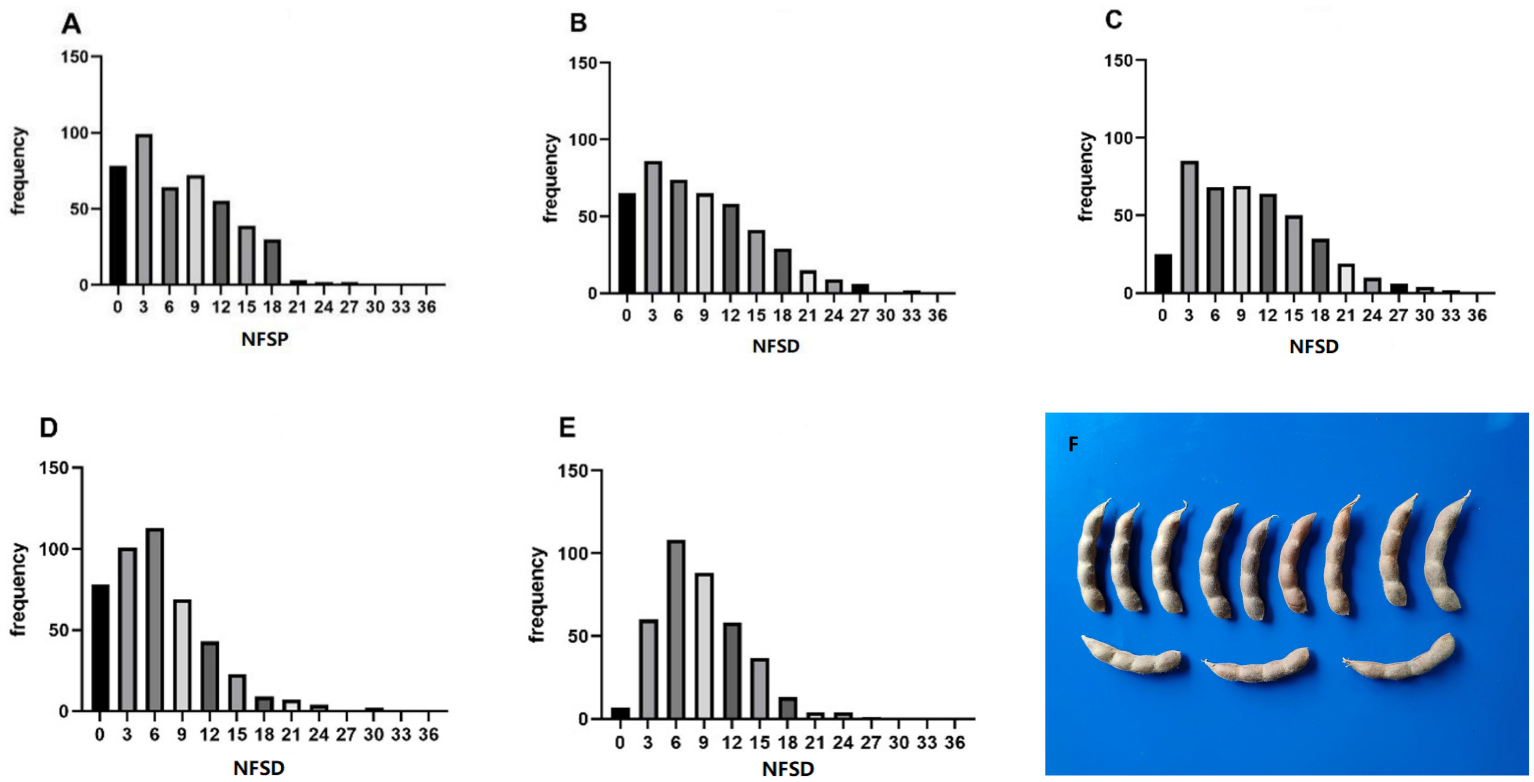
Frequency distribution of NFSP in five environments. The frequency distribution of the soybean NFSP phenotype in the five environments of **(A)** 21AC, **(B)** 21XY, **(C)** 23AC, **(D)** 23QQ, and **(E)** 23WD. **(F)** The morphological photo of four-seed pods. NFSP, number of four-seed pods.

The statistical results of phenotypic data of the soybean NFSP in the GP under five environments are shown in **Figure 2**. In the frequency distribution of NFSP in the GP across the five environments, a skewed distribution toward lower values was observed. In the 21AC, 21XY, and 23AC environments, the frequency of NFSP of 3 was the highest, accounting for 22.20%, 19.11%, and 19.45%, respectively, followed by 6, 9, and 12. In the 23QQ and 23WD environments, the frequency of NFSP of 6 was the highest, accounting for 25.06% and 28.42%, respectively, followed by NFSP values of 3 and 9.

The descriptive analysis of NFSP in the GP under five environments is presented in **Table 1**. The absolute values of kurtosis of the population in different environments were close to 1, indicating that the NFSP phenotypic data of the GP basically followed a continuous variation distribution. The coefficient of variation was relatively large in each environment, and the genotype variance was extremely significant, suggesting that there was genetic variation in NFSP among different varieties within the GP. The means, standard deviations, and ranges varied significantly among different environments, and the genotype by environment interaction effect was extremely significant, indicating that NFSP had different expressions in different environments, and different QTNs and genes could be identified in different environments.

**Table 1:** Statistical analysis of four-seed pod numbers from GP in five environments.

Environment	Mean	SD	Coefficient of variation %	Min	Max	Range	Skew	Kurt
21AC	6.58	5.89	89.62	0.00	35.00	35.00	0.92	0.89
21XY	7.64	6.67	87.24	0.00	32.00	32.00	0.82	0.18
23AC	9.14	6.73	73.57	0.00	32.00	32.00	0.72	0.14
23QQ	5.71	5.51	96.62	0.00	35.00	35.00	1.63	4.09
23WD	7.71	4.61	59.79	0.00	25.00	25.00	0.87	0.85

GP, germplasm population.

The broad-sense heritability of NFSP was higher in multiple environments (**Table 2**), indicating that most genes controlling NFSP could be stably expressed in different environments, while a few genes were differentially expressed among environments.

**Table 2:** Variance analysis and heritability of four-seed pod numbers.

Source	*DF*	*SS*	*MS*	*F*	*Pr* > *F*	*σ* ^2^
E(r)	10	325.526	32.553	1.36	0.1928	
E	4	8,236.062	2,059.016	85.97	<0.0001	
G	454	111,474.39	245.538	10.25	<0.0001	12.421
E * G	1,705	119,204.369	69.915	2.92	<0.0001	15.357
Error	4,318	103,412.378	23.949			23.949
Total	6,491	343,364.569				
*h* ^2^						0.727

### 3.2 QTNs associated with NFSP in soybean

A total of 89 QTNs controlling NFSP were detected on 20 chromosomes through seven methods in the mrMLM package, with a phenotypic contribution rate ranging from 0% to 11.47% (**Supplementary Table S3**). The mrMLM, FASTmrMLM, FASTmrEMMA, pLARmEB, pKWmEB, ISIS EM-BLASSO, and 3VmrMLM methods detected 31, 21, 9, 29, 33, 30, and 22 QTNs, respectively. Among them, the pKWmEB method detected the largest number (33 QTNs), while the FASTmrEMMA method detected the fewest number (nine QTNs). A total of 73 QTNs related to NFSP were detected in a single environment by applying the mrMLM, FASTmrMLM, FASTmrEMMA, pLARmEB, pKWmEB, ISIS, and EM-BLASSO methods. In the 21AC, 21XY, 23AC, 23QQ, and 23WD environments, 15, 18, 14, 22, and 7 QTNs were detected, respectively. Further analysis revealed that 34 QTNs related to soybean NFSP could be co-located by multiple methods or in multiple environments (**Table 3**). Among them, five QTNs were detected in multiple environments by multiple methods: AX-90314196 was detected in the 21XY and 23AC environments by methods mrMLM, ISIS EM-BLASSO, and IIIVmrMLM, and AX-90322106 was detected in the 21AC and 23QQ environments by methods pLARmEB, pKWmEB, and ISIS EM-BLASSO. AX-90435702 was detected by methods mrMLM, FASTmrMLM, pLARmEB, pKWmEB, and ISIS EM-BLASSO in the 21AC and 21XY environments; AX-90439914 was detected by methods mrMLM, FASTmrMLM, pLARmEB, and IIIVmrMLM in the 23AC and 23QQ environments; and AX-90487461 was detected by methods mrMLM, FASTmrMLM, FASTmrEMMA, pKWmEB, and ISIS EM-BLASSO in the 21XY and 23AC environments. Two of the six QTNs detected in a single environment were AX-90308949 and AX-90454301. Seven QTNs, AX-90384913, AX-90437943, AX-90458599, AX-90501229, AX-90486841, AX-90513557, and AX-90514080, were detected by five methods, and the other QTN was detected by two or three methods.

**Table 3:** Stable QTNs related to four-seed pod numbers detected by multiple methods or multiple environments.

Method	Environment	Marker name	Chromosome	Position	QTN effect	LOD	*r* ^2^ (%)	Reference
1, 2, 5, 6	21XY	AX-90512118	1	4,439,457	−1.31 to 0.78	3.04–5.92	1.08–4.02	
5, 6	23WD	AX-90523633	2	50,947,645	0.59–0.77	3.01–3.82	2.77–5.06	
2, 4	23AC	AX-90388204	3	47,091,631	0–1.92	3.25–3.26	0–1.43	
4, 5, 6	21AC; 23QQ	AX-90322106	4	5,248,031	−1.09 to 0.7	3.22–7.44	2.13–3.63	Ning et al., 2018
1, 2	21AC	AX-90355916	4	5,276,195	−1.25 to 0.85	3.17–3.65	2.04–4.12	Ning et al., 2018
3, 6	21AC	AX-90497448	5	36,641,584	−2.02 to 1.03	3.41–3.64	1.75–2.14	Liu et al., 2019
4, 5, 7	21AC	AX-90310000	5	41,763,073	1–4.78	0.51–4.53	0.43–2.73	
3, 5, 6	23WD	AX-90385665	9	2,222,201	−1.6 to 1.66	3.39–4.15	2.11–2.9	Liu et al., 2019
1, 6	23QQ	AX-90380549	9	26,246,922	1.01–1.41	3.03–3.88	1.4–2.55	Liu et al., 2019
1, 2, 3, 4, 6	23AC	AX-90514080	9	27,126,425	−1.94 to 0.92	3.21–3.94	1.41–2.64	Liu et al., 2019
1, 2, 4	21AC	AX-90433162	9	41,044,657	−1.82 to 0.81	4.24–7.16	1.74–4.26	Ning et al., 2018 Vieira et al., 2006
1, 2, 4, 5, 6, 7	23AC	AX-90308949	9	45,912,518	1.25–12.29	0.86–7.66	1.74–6.23	
1, 2, 4, 5, 6	21XY	AX-90486841	10	47,767,513	0.85–1.22	3.61–6.16	1.6–4.04	Liu et al., 2011
1, 2, 4, 7	23AC; 23QQ	AX-90439914	10	49,967,144	−1.01 to 9.08	−0.73 to 4.29	1.34–3.25	
1, 2, 4, 5, 6	23AC	AX-90437943	11	2,180,923	0.86–1.09	3.28–4.53	1.55–3.67	Ning et al., 2018
1, 4, 5, 6	23QQ	AX-90314120	11	6,038,751	1.12–1.81	3.14–4.97	0.94–4.89	Sun et al., 2006
1, 5	23WD	AX-90305288	11	7,262,127	−2.39 to 1.6	3.54–5.35	6.6–6.81	Sun et al., 2006 Zhang et al., 2010
5, 6	23QQ	AX-90477061	12	6,109,948	−1.31 to 1.24	4.75–5.25	2.41–3.89	
2, 3, 6	23WD	AX-90414576	12	6,226,984	−1.8 to 0	3.04–3.62	0–2.01	
1, 2, 4, 5, 6	21XY	AX-90458599	13	19,283,704	−1.85 to 1.6	3.12–5.44	3.05–5.03	Tischner et al., 2003
1, 2, 4, 5, 6	23QQ	AX-90513557	13	21,995,904	−1.49 to 0.84	3.42–6.39	1.55–4.55	Tischner et al., 2003
1, 2, 4, 5	21XY	AX-90453898	13	29,844,012	0.84–1.33	3.69–4.58	1.45–3.67	Fang et al., 2017
1, 2, 4, 5, 6	23AC	AX-90384913	13	36,034,579	1–1.49	3.05–4.5	1.86–3.37	
1, 6, 7	21XY; 23AC	AX-90314196	14	3,021,120	−0.99 to 10.95	0.79–3.56	1.54–1.81	
1, 2, 5, 7	21XY	AX-90329712	15	12,203,882	−2.54 to 41.82	−1.58 to 4.36	1.3–5.25	Ning et al., 2018
1, 2, 4, 5, 6	21AC; 21XY	AX-90435702	16	1,543,661	−1.82 to 0.76	3.2–6.4	1.8–6.53	
1, 2, 4, 6	23AC	AX-90326632	16	31,040,785	−1.25 to 0.82	3.11–4.08	1.44–3.38	Tischner et al., 2003 Ning et al., 2018
1, 2, 4, 5, 6	23QQ	AX-90501229	18	17,683,013	−1.46 to 0.92	3.9–5.91	1.83–4.25	
1, 4	23QQ	AX-90410394	19	40,694,436	0.91–0.97	4.6–5.46	2.26–2.82	Ning et al., 2018
1, 2, 3, 5, 6	21XY; 23AC	AX-90487461	20	34,521,486	1.33–3.97	5.08–12.83	6.19–8.85	Li et al., 2008 Jeong et al., 2011
1, 2, 3, 4, 5, 6	21AC	AX-90454301	20	34,958,132	1.46–3	5.41–10.43	5.63–11.47	Li et al., 2008 Jeong et al., 2011

QTNs, quantitative trait nucleotides.

In addition, four QTNs with additive and environmental interaction effects were detected by the IIIVmrMLM method (**Table 4**), and the ratio of phenotypic variation explained by these QTNs ranged from 0.572% to 1.1794%. The main effect of AX-90514080 also reached a significant level (**Table 3**).

**Table 4:** QTN based on the additive by environment interaction effect via IIIVmrMLM method.

Marker	Chromosome	Position (bp)	LOD	add * env1	add * env2	add * env3	add * env4	add * env5	Variance	*R* ^2^ (%)	p-Value	Significance	Reference
AX-90398950	4	38,248,564	7.8689	−0.4376	0.8195	0.366	0.2973	−1.0451	0.4355	1.1794	2.5874E−07	SIG	
AX-90514080	9	27,126,425	3.8134	−0.8767	0.0584	0.0916	0.2861	0.4405	0.2112	0.572	0.00150352	SUG	Liu et al., 2019
AX-90428842	16	714,535	7.4222	−0.2328	0.9509	0.1954	0.0778	−0.9913	0.3971	1.0752	6.8477E−07	SIG	
AX-90329698	18	6,515,078	5.7243	−1.0801	0.0742	0.2831	0.168	0.5548	0.3177	0.8602	2.6766E−05	SUG	

add * env1, add * env2, add * env3, add * env4, and add * env5 represent additive by environment interaction effect under five environments.

SIG, significant; SUG, suggested.

### 3.3 Prediction and haplotype analysis of NFSP-related candidate genes

**Figure 3:**
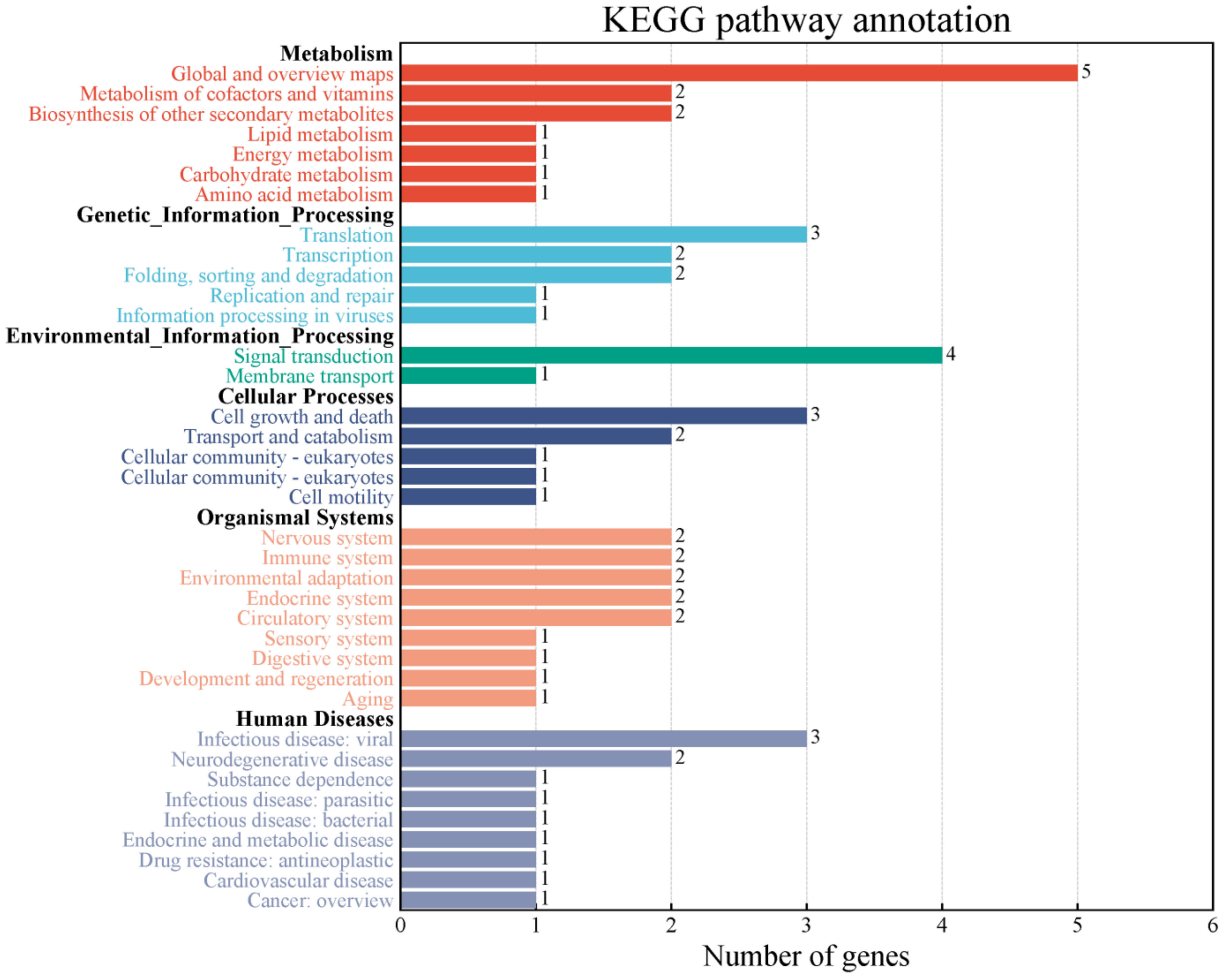
Two-classification summary of genes through KEGG pathway enrichment analysis. KEGG, Kyoto Encyclopedia of Genes and Genomes.

In the decay regions (43 kb before and after) of 34 stable QTNs associated with soybean NFSP, a total of 267 genes were retrieved from the Phytozome website (**Supplementary Table S4**). Further enrichment analysis by the KEGG pathway showed that these genes could be divided into six categories and 37 metabolic pathways (**Figure 3**).

Through literature search and the National Center of Biotechnology Information (NCBI) website (www.ncbi.nlm.nih.gov), 49 genes possibly related to NFSP were further screened by analyzing gene function annotations (**Supplementary Table S5**). Using the resequencing data of 202 germplasm resources, the non-synonymous mutation sites in the coding regions of the above 49 genes were found through the Vcftools software, and the coding region sequence variation of three genes, *Glyma.13G105400*, *Glyma.04G063700*, and *Glyma.04G063100*, were found (**Table 5**). Among them, *Glyma.13G105400* was a DNA-binding protein due to a base mutation resulting in the conversion of alanine to valine. *Glyma.04G063700* changed from methionine to isoleucine, and the functional annotation was the zinc lipoprotein superfamily. *Glyma.04G063100* changed from phenylalanine to leucine, and the functional annotation was the protein kinase superfamily.

**Table 5:** Three candidate genes with significant variation and their mutation sites.

Genes	Marker	Mutation site	Mutation base	Functional annotation
*Glyma.04G063100*	AX-90322106	5,212,739	A–G	Protein kinase superfamily protein
*Glyma.04G063700*	AX-90322106	5,257,832	G–A	RING/FYVE/PHD zinc finger superfamily protein
*Glyma.13G105400*	AX-90513557	22,769,101	C–T	Basic helix-loop-helix (bHLH) DNA-binding family protein

**Figure 4:**
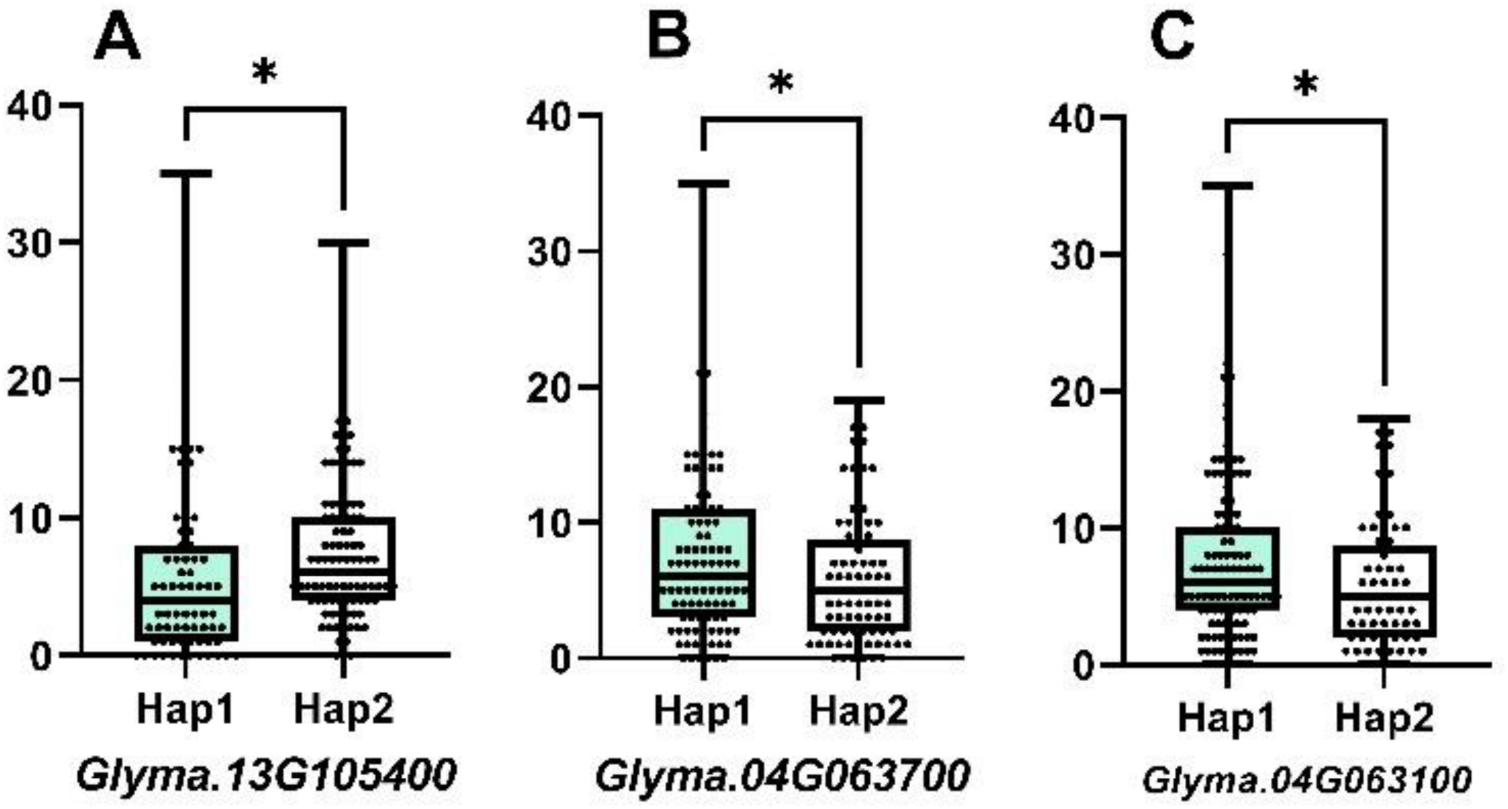
The difference between two haplotypes of three candidate genes. For *Glyma.13G105400*, the mean of Hap1 is 5.803, n_1_ = 76, the mean of Hap2 is 7.606, n_2_ = 104, p-value is 0.0365, and r^2^ = 0.02434. For *Glyma.04G063700*, the mean of Hap1 is 7.571, n_1_ = 105, the mean of Hap2 is 5.845, n_2_ = 84, p-value is 0.0403, and r^2^ = 0.02229. For *Glyma.04G063100*, the mean of Hap1 is 7.504, n_1_ = 113, the mean of Hap2 is 5.735, n_2_ = 68, p-value is 0.0406, and r^2^ = 0.02321.

According to the sequence variation of the coding region, the three genes showed two allelic genotypes, Hap1 and Hap2, forming two haplotypes. Combined with phenotypic data and grouped by haplotype, the difference in NFSP was tested for significance, and there were significant differences in NFSP among haplotypes of each gene, as shown in **Figure 4**. The NFSP value of Hap1 of *Glyma.13G105400*, *Glyma.04G063700*, and *Glyma.04G063100* genes was higher than that of Hap2.

### 3.4 Optimal breeding schemes for NFSP

Using the genotypes and the NFSP phenotypic data of a single plant in five environments (**Supplementary Table S6**) of 455 varieties in the GP, MAS models were established for five environments, and the regression effects of 89 QTNs on a single NFSP reached a significant level in all five environments. In the five environments of 23AC, 21XY, 21AC, 23WD, and 23QQ, the model could explain 50.1%, 55.06%, 52.35%, 44.82%, and 50.56%, respectively, of the phenotypic variation in NFSP (**Supplementary Table S7**).

**Figure 5:**
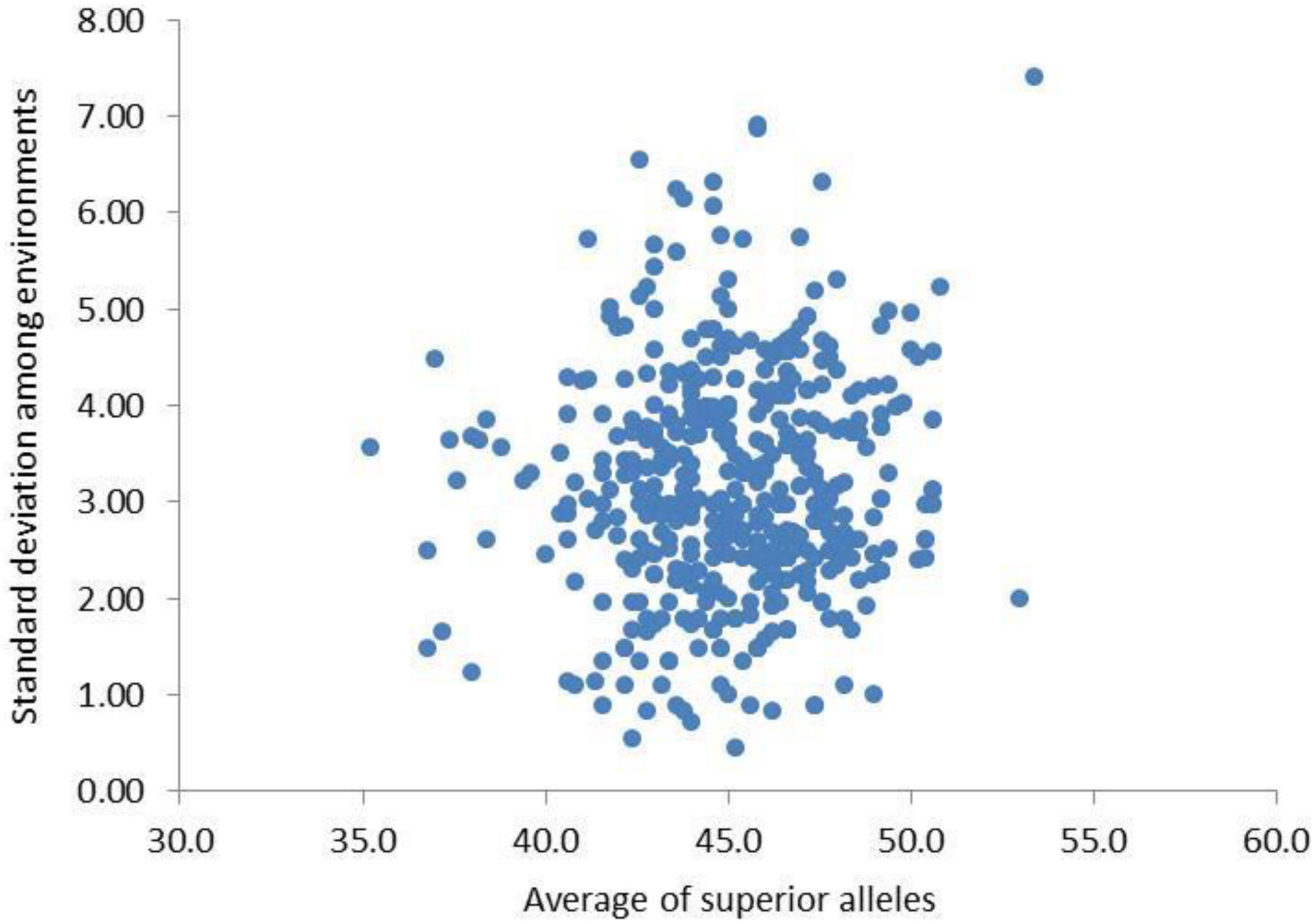
Mean and standard deviation of NSA of 455 varieties in five environments. NSA, number of superior alleles.

Based on the genetic effect values of QTNs in the above MAS model, the synergistic alleles of the two alleles of QTNs were defined as superior alleles. Among the five environments, there was little difference in the range of the number of superior alleles (NSA) in the original population (parents), which were close to normal distribution (**Table 6**). The mean and standard deviation of NSA for each individual varied greatly among different environments, indicating that the NSA of each individual was affected by the environment, which laid the genetic basis of genotype × environment interaction in NFSP (**Figure 5**).

**Table 6:** The frequency of NSA of NFSP in the original population under five environments.

Number of superior alleles	Environment				
	21AC	21XY	23AC	23QQ	23WD
31	0	0	0	0	1
32	0	1	2	1	0
33	0	0	1	1	1
34	0	3	0	2	1
35	0	3	3	4	2
36	6	4	1	4	4
37	3	8	2	13	3
38	7	14	2	13	6
39	5	14	5	18	11
40	19	26	10	33	11
41	24	30	10	35	27
42	24	43	21	44	34
43	41	50	35	39	38
44	44	51	44	50	68
45	52	37	44	50	43
46	43	47	36	32	56
47	43	38	32	25	46
48	26	24	59	33	39
49	27	16	40	18	19
50	29	16	42	16	10
51	25	10	28	6	13
52	11	10	14	6	11
53	9	5	8	7	7
54	9	2	8	3	4
55	3	1	5	0	0
56	3	1	2	1	0
57	1	0	1	0	0
58	0	0	0	0	0
59	0	1	0	0	0
60	0	0	0	1	0
61	1	0	0	0	0

NSA, number of superior alleles; NFSP, number of four-seed pods.

For the single-cross combination of two parents, after seven generations of selection of each breeding scheme, the number of hybrid offspring obtained by each breeding scheme, the genotype values, and the NSA contained are shown in **Table 7**. In the five environments of 23AC, 21XY, 21AC, 23WD, and 23QQ, the range of NSA (minimum to maximum value) of the hybrid offspring obtained by various breeding schemes was higher than that of the parents (original populations), indicating that NSA can be increased after selection by various breeding schemes. In the five environments, the maximum and mean values of the NFSP genotypes of the hybrid offspring obtained by various breeding schemes were higher than those of the parents (original populations), indicating that an individual NFSP could be improved after selection by various breeding schemes.

**Table 7:** NSA and genotype values of the parents and offspring screened by various breeding schemes in five environments.

Environment	Population	Number of individuals	Number of superior alleles	Genotype value			
				Min	Max	Mean	SD
21AC	Original population	455	36~61	0.00	24.52	8.63	4.56
	Progeny-200Ped	8,263	34~67	0.00	43.70	19.71	8.46
	Progeny-200Blk	20,657	36~66	0.00	45.99	18.09	7.81
	Progeny-500Ped	20,657	36~65	0.00	47.53	19.87	8.52
	Progeny-500Blk	20,675	36~66	0.15	47.05	18.30	7.90
	Progeny-800Ped	33,051	35~67	0.00	47.67	19.93	8.52
	Progeny-800Blk	20,657	35~65	0.00	46.45	18.35	7.91
21XY	Original population	455	32~59	0.00	26.59	7.62	5.13
	Progeny-200Ped	8,263	39~64	2.66	44.74	20.77	5.79
	Progeny-200Blk	20,657	36~64	2.72	42.66	19.83	5.57
	Progeny-500Ped	20,657	39~64	2.77	44.02	20.94	5.85
	Progeny-500Blk	20,657	38~64	3.39	43.06	20.28	5.63
	Progeny-800Ped	33,051	38~64	2.60	48.57	21.06	5.79
	Progeny-800Blk	20,657	38~63	1.80	42.99	20.33	5.65
23WD	Original population	455	31~54	0.00	17.06	7.61	3.25
	Progeny-200Ped	8,263	38~61	1.59	26.93	14.04	3.15
	Progeny-200Blk	20,657	38~63	1.42	25.16	13.90	3.14
	Progeny-500Ped	20,657	36~62	3.04	28.59	14.06	3.15
	Progeny-500Blk	20,657	38~62	1.85	26.91	14.11	3.19
	Progeny-800Ped	33,051	37~63	1.31	26.68	14.09	3.16
	Progeny-800Blk	20,657	38~62	2.99	27.27	14.15	3.17
23QQHE	Original population	455	32~60	0.00	20.14	5.66	3.99
	Progeny-200Ped	8,263	39~65	5.04	30.99	16.82	3.79
	Progeny-200Blk	20,657	36~66	1.97	30.53	16.20	3.88
	Progeny-500Ped	20,657	37~67	3.68	32.48	16.85	3.82
	Progeny-500Blk	20,657	39~66	2.79	34.65	16.46	3.89
	Progeny-800Ped	33,051	37~69	3.74	32.73	16.89	3.84
	Progeny-800Blk	20,657	37~66	3.80	31.35	16.52	3.86
23AC	Original population	455	32~57	0.00	22.54	8.97	5.19
	Progeny-200Ped	8,263	34~64	0.00	42.87	17.16	5.43
	Progeny-200Blk	20,657	37~66	0.00	40.29	16.97	5.21
	Progeny-500Ped	20,657	35~68	0.00	43.32	17.27	5.46
	Progeny-500Blk	20,657	34~65	0.00	43.29	17.31	5.31
	Progeny-800Ped	20,657	35~64	0.00	44.99	17.23	5.51
	Progeny-800Blk	20,657	36~65	0.00	43.48	17.27	5.29

NSA, number of superior alleles.

With the increase of F2 population size, the number of individuals selected by the pedigree method increased, but there was no effect on the number of outstanding offspring individuals selected by the bulk selection method. At the small F2 population size (200), the number of individuals selected by the bulk selection method was more than that by the pedigree method. At a large F2 population size (800), the number of individuals selected by the pedigree method was more than that by the bulk selection method. For the same F2 scale scheme, the standard deviation of the NFSP of the offspring from the pedigree method and the bulk selection method was quite different. For the 21AC, 21XY, and 23AC environments, the standard deviation of the NFSP of the offspring from the pedigree method was higher than that of the bulk selection method, indicating that the variation of the offspring of the pedigree method was better than that of the bulk selection method in these three environments. However, for 23QQ, the standard deviation of the NFSP of offspring treated by the pedigree method was lower than that of offspring treated by the bulk selection method, indicating that the variation range of offspring treated by the bulk selection method was better than that treated by the pedigree method in the 23QQ environment.

The distribution of the number of individuals of each NFSP type in the selected offspring lines of each breeding program in each environment is listed in **Supplementary Table S8**. The distribution of NFSP genotype values of the selected offspring in each breeding program all presented a normal distribution. For each environment, based on the principle that the highest genotype value is the best breeding goal, the cross combinations and their selection methods with the genotype values distributed in the highest two intervals were determined as the best breeding scheme.

**Figure 6:**
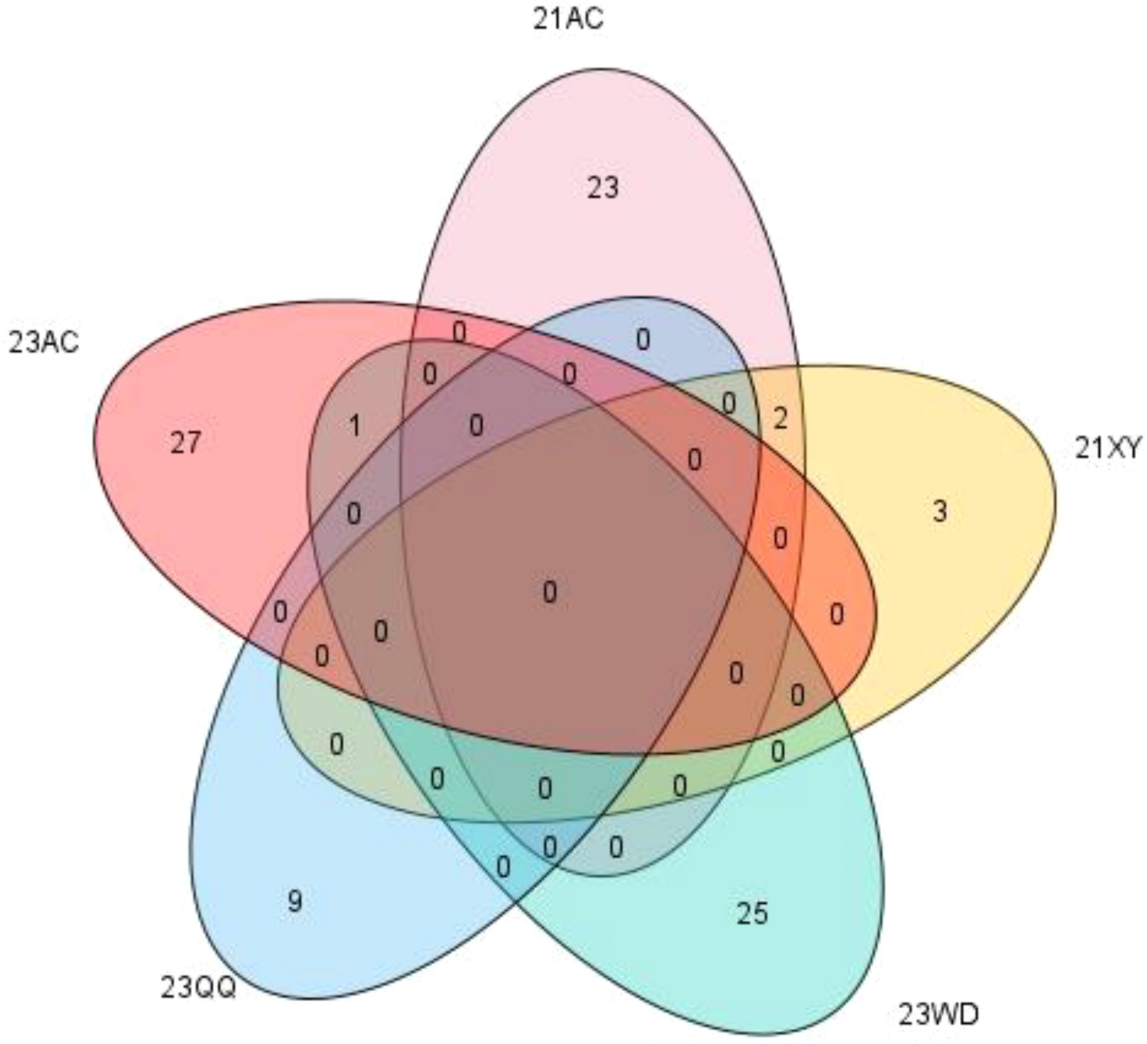
The number of hybrid combinations selected for different environments.

In the 21AC, 21XY, 23WD, 23QQ, and 23AC environments, the breeding goals were determined as NFSP values of over 43, 43, 25, 31, and 40, respectively. A total of 153 schemes were selected from five environments, including 40, 9, 48, 14, and 42 breeding schemes selected from 25, 5, 26, 9, and 28 hybrid combinations, respectively (**Supplementary Table S9**). Except for the CX094/CX246, CX097/CX426, and CX097/CX448 combinations, all hybrid combinations were only selected in one environment (**Figure 6**).

## 4 Discussion

### 4.1 Genetic characteristics of soybean NFSP

Extensive studies have been conducted on the genetic characteristics of pod number-related traits in different genetic populations of soybeans. The number of pods per plant in the RIL population derived from Charleston × Dongnong 594 showed a normal distribution (Chen et al., 2007). The number of pods per plant in the RIL population derived from Bogao × Nannong94–156 presented a normal distribution at all growth stages (Zhang et al., 2010). In this study, the NFSP of the germplasm population consisting of 455 varieties showed a skewed continuous distribution in five environments. This is due to the different genetic bases of the natural germplasm population and the RIL population. The former is composed of varieties selected by artificial or natural selection, while the latter is formed by the genetic recombination of allelic variations carried by two of four parental varieties.

NFSP in soybean is a quantitative trait controlled by multiple genes. In the present research, a total of 89 QTNs were detected in five environments, namely, 21AC, 21XY, 23AC, 23QQ, and 23WD, with the phenotypic contribution rate of individual QTNs ranging from 0% to 11.47%, indicating that NFSP is controlled by both major and minor genes. This is similar to the phenotypic contribution rate of 23 NFSP QTLs detected in the four-way recombinant inbred line population, which ranged from 2.24% to 10.8% (Liu et al., 2019). Notably, among all the QTNs identified in this study, AX-90454301 (Gm20:34958132) had a phenotypic contribution rate of 5.63%–11.47%, which is considered a major gene and is located in close proximity to the reported Ln gene for four-seeded pods (Gm20: 35827672–35830107) (Jeong et al., 2012) (**Table 4**).

The heritability of pod number per plant in a single environment of the RIL derived from BARC-8 and Garimpo was 74.29%, and the genotype × environment interaction effect reached a significant level (Vieira et al., 2006). The heritability of NFSP in different sites in a single environment of the four-way recombinant inbred line population derived from (Kenfeng 14× Kenfeng 15) × (Hei Nong 48× Kenfeng 19) was 21.25%–53.74% (Liu et al., 2019). In this study, the genotype × environment interaction variance of NFSP in five environments reached a significant level, and the heritability was 72.7%, which was similar to the traits related to pod number in the RIL populations in previous studies, indicating that NFSP not only had a stable genetic basis in multiple environments but also had specific genetic basis in different environments. In this project, a total of 89 QTNs were detected in five environments, and among all QTNs, five could be repeatedly detected in more than two environments (**Table 4**), indicating that these QTNs were multi-environment general QTNs, while the remaining 84 QTNs belonged to environment-specific QTNs.

### 4.2 Co-localization analysis of QTNs for NFSP in multiple genetic backgrounds

Among the 34 QTNs repeatedly identified in multiple environments and by methods in this study, 19 were co-located with genomic regions related to pod number identified in previous studies (**Table 4**). Specifically, AX-90322106, AX-90355916, AX-90437943, AX-90329712, and AX-90410394 were located in the study by Ning et al. (2018). AX-90497448, AX-90385665, AX-90380549, and AX-90514080 were co-located with QTLs identified by Liu et al. (2019). AX-90486841 was within the interval identified by Liu et al. (2011). AX-90314120 was within the interval identified. AX-90458599 and AX-90513557 were co-located with the intervals identified by Tischner et al. (2003). AX-90433162 was detected in the studies by Ning et al. (2018) and Vieira et al. (2006). AX-90305288 was detected in the studies by Sun et al. (2006) and Zhang et al. (2010). AX-90453898 was co-located with the results of Fang et al. (2017). AX-90326632 was repeatedly detected by Tischner et al. (2003) and Ning et al. (2018). AX-90487461 and AX-90454301 were located in the studies by Li et al. (2009) and Jeong et al. (2011). The remaining 15 QTNs were newly discovered in this study and require further research for validation.

### 4.3 Analysis of NFSP candidate genes in soybean

This study predicted a total of three genes related to NFSP in soybeans, namely, *Glyma.13G105400*, *Glyma.04G063700*, and *Glyma.04G063100*. Among them, the function annotation of the *Glyma.13G105400* gene is a basic helix-loop-helix (bHLH) DNA-binding family protein. It activates DNA transcription factors and participates in the binding of specific DNA sequences, which may be related to seed meiosis. The function annotation of the *Glyma.04G063100* gene is a protein kinase superfamily protein. The protein kinase superfamily is widely present in flowering plants and usually plays a regulatory role in metabolism and cell division. The function annotation of the *Glyma.04G063700* gene is RING/FYVE/PHD zinc finger superfamily protein. In *Arabidopsis thaliana*, this gene shows negative regulation when expressed and is related to photoperiod, flowering time, or degree regulation. Based on the above analysis, it can be considered that *Glyma.13G105400*, *Glyma.04G063700*, and *Glyma.04G063100* can be used as target genes for the next step in research. Their specific functions can be verified through experiments related to functional validation, contributing to breeding programs.

### 4.4 Studies on simulating breeding

In traditional breeding, it is based on the phenotypes of target traits to select parents and screen individuals of the hybrid combination. This entire breeding cycle often takes several years, and it is uncertain whether an ideal variety can be selected. Simulation breeding, in contrast, utilizes computer models and algorithms to predict the genotype values of the offspring from various genetic combinations based on the genotypes of molecular markers associated with the target traits and to simulate and analyze various environments and breeding schemes, thereby enabling the selection of hybrid combinations and breeding schemes (Li et al., 2025). Zhang et al. (2022) introduced the use of the Blib platform to handle various complex genetic effects and genetic models. You et al. (2022) and Wang et al. (2023) respectively conducted predictions on flaxseed and wheat hybrid combinations. This study applied the ISI module (Li et al., 2025) to simulate breeding schemes processed by the pedigree method and the mixed-selection method for single-cross combinations. Among all 455 parent combinations, 153 breeding schemes from 70 combinations were selected. Through these schemes, individuals with multiple NFSP genotype values in various environments could be screened. During the analysis, two points were considered. First, the molecular marker effect models should be constructed for each environment using all QTNs. For multi-environment experiments, due to the genotype × environment interaction effect, the genetic effect of each allele variant of QTNs varies in different environments. When the contribution to the phenotype is small, QTNs will not be detected. To evaluate the effects of all QTNs in different environments, molecular marker effect models should be constructed for each environment using all QTNs. Therefore, the first step in the simulation study of this research was to construct molecular marker effect models in five environments, which could explain 44.82% to 55.06% of the NFSP variation, also indicating the influence of the QTN × environment interaction effect on the contribution rate of the phenotype. Second, the selection of the optimal hybrid combination should be based on the distribution of the breeding values of the simulated offspring population. Breeding value is the sum of the effects of all allelic variations at the gene loci of an individual’s target trait and thus can be used as the basis for individual selection. In this study, since all individuals in the germplasm resource population were used as parents for the simulation study, the number of hybrid combinations was large, and the scale of the selected offspring population was also large. When organizing the simulation results, the frequency distribution of the breeding values of all selected offspring was first conducted, and then the selection range of the breeding values was determined based on the characteristics of the breeding trait and the scale of the breeding population, thereby screening excellent hybrid combinations. In this study, the frequency distribution of the offspring individuals selected by each breeding scheme in each environment was conducted, and then the individuals categorized into the two highest groups were selected as the hybrid combinations based on the requirements of the NFSP breeding trait and the scale of the breeding population.

## 5 Conclusion

In this study, a genome-wide association analysis was conducted on the number of four-seeded pods of a germplasm resource population comprising 455 soybean varieties under five environments. A total of 89 significant QTNs were identified, and three genes related to the formation of NFSP in soybeans, namely, *Glyma.13G105400*, *Glyma.04G063700*, and *Glyma.04G063100*, were identified. A molecular-assisted selection model containing 89 QTNs was constructed for each of the five environments, and a total of 153 breeding programs were selected. The results of this study lay the foundation for dissecting the genetic mechanism of soybean pod formation and for cultivating high-yielding varieties.

## Data Availability

The original contributions presented in the study are included in the article/**Supplementary Material**. Further inquiries can be directed to the corresponding authors.
